# Association between low back pain and various everyday performances: Activities of daily living, ability to work and sexual function

**DOI:** 10.1007/s00508-019-01542-7

**Published:** 2019-09-06

**Authors:** Igor Grabovac, Thomas Ernst Dorner

**Affiliations:** Department of Social and Preventive Medicine, Centre for Public Health, Medical University of Vienna, Kinderspitalgasse 15/1, 1090 Vienna, Austria

**Keywords:** Low back pain, Everyday functioning, Activities of daily living, Work ability, Sexual function

## Abstract

Low back pain (LBP) is a widely prevalent chronic pain disorder associated with a high burden on individuals and society. In the subjective perception of patients with LBP, probably the most important health outcomes associated with LBP are those that effect everyday performance. Such outcomes include reduction in activities of daily living (ADL), in work ability (WA), and in sexual function. This narrative review aimed to (1) examine the association between LBP and the three mentioned outcomes of everyday performance, (2) to explain possible mediating factors promoting these associations, and (3) to discuss possible implications for treatment and rehabilitation. Studies have shown that LBP can generate anxiety of movement leading to movement avoidance (fear-avoidance beliefs), which may lead to deconditioning and further increasing problems with ADL, WA and decreasing sexual function. Furthermore, common mental disorders, such as depression, anxiety, and stress-related disorders, which also often co-occur with LBP can lead to adverse effects on everyday performance and vice versa, can be the consequence of such problems and aggravate LBP. Although there is no universally accepted treatment modality that fits every patient with LBP, physical training, comprehensive patient education, and workplace or home modifications have been shown to be able to interrupt the mutual influence between LBP and the described mediating factors, and have a beneficial effect on ADL, WA, and sexual function. For this, a multidisciplinary approach is necessary which includes multiprofessional care teams, participation of the patients, and involvement of different settings, such as workplace, home, and physical training facilities.

## Introduction

Low back pain (LBP) is one of the most prevalent conditions worldwide, with the results of the Global Burden of Disease (GBD) study in 2010 reporting a global point prevalence of 9.4% [1]. The World Health Organization further reported that up to 70% of the population in industrialized countries will experience non-specific LBP (i.e. without a confirmed pathoanatomical cause as opposed to specific LBP that may be linked to intervertebral disc damage, such as herniation or fractures, vertebral infections, cancer including bony metastases and spondylarthritis [2]) in their lifetime [3]; however, there is increasing evidence showing that LBP prevalence is also increasing in the developing world [4]. The prevalence of LBP seems to increase with age, with the peak being between the ages of 35 and 55 years [3]. LBP has a strong tendency to become chronic [5] and is among the most commonly reported localizations for chronic pain issues, with some reports showing global prevalences of almost 20% in people aged between 20 and 59 years [6]. The results of the Austrian Health Interview Study, showed that 25% of respondents in this nationally representative survey reported chronic pain in any body site and the 1‑year period prevalence of chronic LBP was 10% of the adult population [7]. Moreover, studies of epidemiological monitoring of LBP in the USA have reported a rising trend across age groups and in both men and women [8, 9].

A broad variety of factors are involved in the development of LBP and often limited effects in treatment lead to LBP being associated with very many detrimental health outcomes, such as disability and overall limited mobility, poorer self-reported health, lower quality of life and depression as well as more workplace absenteeism [3, 10]. These issues as well as the growing prevalence put LBP as a major public health problem, associated with increasing costs for social systems. Results of a recent Austrian study looking into societal costs linked to chronic pain issues reported overall annual costs of 10,191 Euros per patient, with inpatient rehabilitation, and out-of-pocket costs being identified as the two most expensive costs factors, the latter being also the conclusion of a 2008 Austrian study [11, 12].

Patients with LBP often report issues with routine functioning and participating in daily activities, with impairments in interpersonal relations and community life being especially important for patients with LBP. An Austrian study reported that in patients with chronic LBP the strongest association with health satisfaction was not needing medical treatment to function in daily life. In men with chronic LBP additionally satisfaction with sex life and satisfaction with work capacity strongly determined health satisfaction, while in women such a determining factor was satisfaction with living conditions [13]. This suggests that functional independence, work ability and sexual function are essential and probably the most important health outcomes of people with LBP in their own perception. Aging, however, may also affect personal physical and psychological resources, changing the importance of these functions during the life span.

With the apparent growing public health issues connected to LBP and the growing number of publications, often with conflicting results, this study aimed to summarize the current knowledge on functional performance in people with LBP through its influence on activities of daily living, work ability and sexual function in a narrative review. Furthermore, it was the aim to reveal and discuss potential factors mediating the association between LBP and those three measures of everyday performance.

## Methods

We conducted a narrative review to provide an overview of the topic. Both authors independently searched two databases (Google Scholar and PubMed). We screened for functional performance including activities of daily living, work ability and sexual function and the associations with LBP. Furthermore, we reviewed the reference lists of included articles. It was of interest to see to what degree LBP influences the three outcomes of everyday performance that were of interest: (1) associations of LBP on activities of daily living, (2) influence of LBP on workability and (3) effects of LBP on sexual function.

## Results

### Associations between LBP and activities of daily living

Activities of daily living (ADL) are various functional activities that may range from basic ones, such as walking or bending, to more complex activities (also called instrumental activities of daily living, IADL), such as cooking, bathing or getting dressed, in other words activities which enable independent living [14, 15]. There seems to be a consensus across studies that LBP is associated with problems in ADL. An Austrian study in the general population aged 65 years and over found a clear association between LBP and problems in ADL with an odds ratio (OR) 2.01 (95% confidence interval, CI 1.57–2.57) and IADL with OR 2.17 (95% CI 1.82–2.59), adjusted for sociodemographic, lifestyle and health-related parameters. Another Austrian cross-sectional study of older adults with and without osteoporosis, osteoarthritis and chronic back pain using a nationally representative dataset reported that doing heavy housework, bending or kneeling, climbing stairs up and down without walking aids and walking 500 m without a walking aid were the most problematic ADLs in all groups [16]. Interestingly, people with chronic LBP reported a much larger number of problematic ADLs compared to those with other musculoskeletal diseases or without them [16].

A Thail cohort study (*N* = 42,785; 80% aged between 30 and 50 years) showed that 30% of the cohort participants reported LBP, where approximately 6% of the cohort reported difficulties in bending, 3.1% had difficulties in walking a 100 m, 2.2% could not climb stairs, and a further 2.9% had problems when dressing. This longitudinal cohort study reported a time-dependent increasing gradient in the functional limitation across all activities [17]. This study provided interesting results not only due to its longitudinal design but also gave insights into the LBP problems occurring in middle income level countries, which are seldom presented in research [4]. As mentioned in the introduction, the high prevalence of LBP is a known public health issue in industrialized countries [3]; however, longitudinal studies investigating LBP as a disability factor are rare. Some studies however, showed that LBP is an independent factor that worsens the self-reported disability level and makes ADL much harder for people who are already living with disabilities. For example, results from the Women’s Health and Aging Study (*n* = 1002) showed that 42% of older women with disability reported LBP. After multivariate adjustments, women with severe back pain were 3–4 times more likely to report difficulties with light housework or shopping as well as having an increased likelihood of issues with various mobility tasks [18]. Results of this study need to be interpreted in the light of the study participants, namely older women (30% of participants older than 85 years) who were already living with a serious disability. Similar results came from a cohort of patients living with rheumatoid arthritis (RA). In a study population of 281 patients with RA, 53.4% reported LBP over a 6-month period. Those patients who reported experiencing LBP presented with significantly higher disability in ADL compared to RA patients without LBP. This study found a moderate effect of LBP, which was enough to demonstrate a clinical relevance of LBP comorbidity in this patient group [19]. Some studies looked into patient groups with an objectively confirmed etiology of reported LPB. A Turkish study investigated differences in ADL in patients with LBP resulting from lumbar disc herniation between those who received conservative treatment and those who underwent surgery. Prior to treatment they found that patients in both groups reported similar issues, mostly problems with prolonged standing, lifting weights and socializing. At follow-up (3 months following treatment) there seemed to be no differences in ADL that the patients had problems with; however, it is important to note that the patients who received conservative treatment reported worsening in terms of experienced pain [20]. These results need to be interpreted with caution as the study did not report on surgical or conservative treatment protocols, post-surgery complications, physiotherapy or occupational therapy that the study patients underwent.

Studies of both the general population as well as populations of patients with other chronic illnesses or disabilities reach a consensus that LBP causes problems in functional capacity and performing ADL [21–23]. The reason for this association may be in the deconditioning syndrome (complex process of physiological changes due to periods of inactivity [24]), which has been reported in substantial numbers of patients with chronic LBP issues [25–27]. Furthermore, LBP and ADL deficits do not only occur together very often. If chronic pain and ADL deficits coincide, they work synergistically towards an adverse outcome. Subjects affected by both ADL deficits and chronic pain showed a strong synergistic effect towards health care utilization. This means that healthcare utilization was much higher than could be expected from the mere addition of the health care utilization due to ADL deficits plus health care utilization due to chronic pain [28].

### Association between LBP and work ability

The LBP is the most recurrent of all chronic conditions experienced by the working population and is one of the leading causes of disability and absence at work associated with high socioeconomic impact. As mentioned in the introduction, LBP is associated with very high costs, with indirect costs (which include loss of productivity or loss of working days) representing more than two thirds of the total costs [29]. The results of two US national surveys showed that more than 100 million working days are lost each year due to LBP [30, 31]. In Austria musculoskeletal problems accounted for the highest number of sick leave days in 2017, with LBP being one of the most commonly reported problems [11, 32]. One of the reasons why LBP has a strong influence on working ability and loss of productivity is the high prevalence in adulthood during the most economically productive ages (30–60 years) [3, 33].

Epidemiological analyses point to several work-related activities (lifting or pushing weights, vibration exposure, various ergonomic issues) that may be the cause of LBP or at least increase the risk for recurrence [34, 35]. This leads to increased disability, absenteeism and employee turnover. Therefore, not surprisingly, LBP ranks among the most expensive medical conditions [33]. The LBP seems to be very prevalent among healthcare workers, with the 1‑year prevalence being reported between 45% and 77%, which is more compared to other occupations [36].

A study by Nordstoga et al. of 165 patients with non-specific LBP seeking primary physiotherapy reported that higher work ability was associated with less disability, less pain and higher quality of life [37]. Increased psychological distress caused by LBP and the number of pain sites were associated with higher disability, more pain, and lower quality of life. After 3 months follow-up improvements in work ability showed significant associations with improvement in disability, pain and quality of life [37]. These results support the notion that improving the patients’ ability to work will have farther reaching and overall effects on multiple health outcomes. Interestingly, the same study reported that reduced psychological distress was only associated with improvements in pain but not work ability. Another study from Nordstoga et al. further showed that fear of pain reoccurrence leads to avoidance of certain movements, called fear-avoidance beliefs (FAB), which were associated with both levels of reported disability and work ability [38]. Moreover, a Finnish cross-sectional study of 219 female healthcare workers with non-specific LBP investigating pain level, physical functioning and ability to work reported that the strongest associations of better work ability were lower work-induced lumbar exertion, better perceived work recovery, lower depression and lower work-related FABs [39].

Interesting results come from a Japanese study by Tsuboi et al. that looked into the associations between presenteeism and FAB among workers with LBP providing care for old people [40]. Presenteeism is the opposite of a more well-known concept of absenteeism and may be defined as workers being on the job but because of illness or other medical conditions, not fully functioning [41]. Interestingly, presenteeism is also associated with higher socioeconomic burden with some studies reporting the costs being 2–5 times higher and losses of productivity being 2–3 times higher than those associated with absenteeism [42]. A recent Japanese study reported the costs of absenteeism of US$520 and presenteeism at US$3055 per patient per year [43]. The adjusted model of this study showed that higher scores of kinesophobia (fear of movement) resulting from LBP were associated with higher presenteeism and there were significant associations between kinesophobia scores and all the work ability subscales (e.g. time management, mental interpersonal demands, physical demands and output demands).

### Association between LBP and sexual function

Sexual function and sexuality have an effect on patients’ overall quality of life; however, questions regarding sex, sexual function or practices are often overlooked by researchers and practitioners but also patients and study participants [44, 45]. For example, an Australian study found that nearly half of the respondents using the Oswestry Disability Index, a widely used instrument for assessing chronic LBP, did not complete the section specific to sex life [46]. The study reported that there are widespread anecdotal beliefs that questions on sex life are inaccurately answered and that the mere presence of a question on sexuality may repel some participants from filling it out; however, this was not found to be accurate in this study and other studies that reported an overall response rate to sexuality questions of 97%. The study also found that those participants who responded to the question on sexuality did so accurately [46]; however, studies remain scarce in this respect.

An Austrian study, carried out within the framework of regular health check-ups in supposedly healthy people, found subjects who reported sexual dissatisfaction had a threefold higher chance of being affected by joint and muscle pain in men and a 2.64 times higher chance in women [47]. An early Swedish study (published in 1981) in 35 male and 25 female participants investigated various sexual outcomes and chronic back pain [48]. Almost half of both male and female participants reported an overall reduction in intercourse with 37% of men reporting a decrease in erections and 23% of men and 28% of women reporting a decrease in the frequency of orgasms. Coital positions also changed in frequency before and after the onset of LBP in both groups. Half of the male respondents and 80% of women named fatigue as a reason that prevents them fully enjoying sex. Overall, 54% of men and 52% of women felt the general satisfaction with sex decreased after the onset of LBP. The authors hypothesized about the underlying effect of depression as a common psychiatric comorbidity of LBP but also that painful muscle hypertonia may be a result of somatic conversion [48]. Results of this study were confirmed in subsequent studies that consistently showed reduction in sex frequency following LBP as well as more or worsening pain as a result of coitus as well as discomfort and problems in finding the appropriate sexual position [49–52]. Results from a more recent Iranian study comparing 702 men and women with LBP with 888 healthy controls showed that the prevalence of sexual issues in female patients with chronic LBP was 71.1% while 36.8% of women without LBP had corresponding results. Erectile dysfunction was reported by 59.5% of men with LBP, compared to 24.5% in healthy men [52]. Better sexual functioning in both males and females was associated with younger age, shorter duration of LBP, lower body mass index (BMI), higher education level, unemployment, being physically active, being on shorter sick leave, lower pain and disability, higher family income and lower depressive and anxiety symptoms and better psychological functioning [52]. Results of these studies led to a hypothesis that sexual issues are not only psychological but may be also mechanical.

To elucidate the spinal movements in healthy males, a study by Sidorkewicz and McGill made a biomechanical analysis of spine movements and postures during coitus [53]. Based on the measurements, recommendations on sexual positions for males with LBP were made. A rear entry position where the female is in the quadruped position supporting her upper body with her elbows is the most recommended sexual position for flexion-intolerant men. Side lying positions were least recommended. For patients who were motion-intolerant, coital movements from spine dominant to hip dominant are recommended [53]. This study, however, did not take potential problems of the female partner into account nor did it investigate spinal motions in non-heterosexual couples or sexual activities outside vaginal intercourse. Further investigations with more inclusivity and diversity in sexual positioning, practice and sexual orientation of couples included should be done. A systematic review in musculoskeletal pain and sexual functioning in women, albeit not focusing specifically on LBP, concluded that there is still a knowledge gap in the effects of musculoskeletal disorders and sex related outcomes; however, fatigue, medication use and relationship adjustment were found to affect sexuality in women as much as chronic illness [54]. Interestingly a French study found that women with LBP reported greater reduction in coitus frequency, more discomfort and more overall interference in sexual lives compared to women with neck pain [55]. Countrary to the biomechanical analysis recommendations by Sidorkewicz and McGill [53], Rosenbaum suggests the side lying position as most appropriate for women with LBM [54].

In a study including 742 Iranian patients with chronic LBP the mediation effect of sexual functioning on pain and depression was investigated. The study confirmed that in both men and women depressive symptoms showed a significant association with pain intensity and that both models were significantly mediated by sexual functioning, with a medium to large effect in men and medium effect in women [56].

## Discussion

The occurrence of LBP is a multifactorial, debilitating and highly prevalent condition that creates huge socioeconomic burdens on individuals as well as the healthcare systems and society as a whole. This narrative review shows that there is a clear consensus of the effects of LBP on various aspects of functional ability, especially in reduction of ADL, work ability and sexual functioning. Interestingly, the results are relatively homogeneous even across different countries (high and middle to low incomes), cultures and healthcare systems, contributing to the findings that suggest LBP should not be considered as just a problem of industrialized countries.

### Factors mediating the association between LBP and everyday performances

**Fig. 1:**
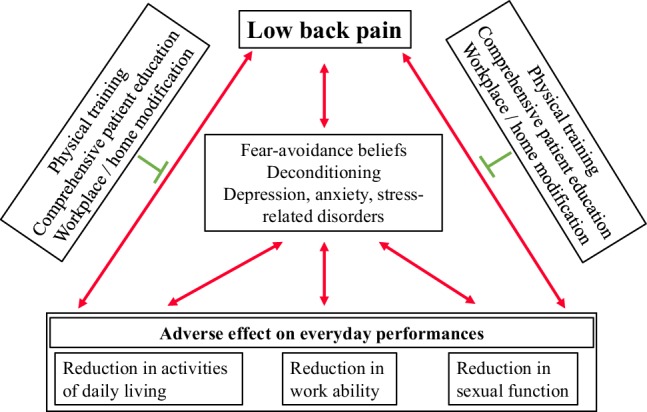
Mediating factors between low back pain and adveres effects on everyday performances

This review also points to the possible mediating factors explaining the reduction in ADL, work ability and sexual function, as shown in Fig. 1. All of the reviewed studies showed that chronic LBP is followed by a level of kinesophobia, which leads patients to FAB and avoid certain movements and activities. Higher scores of FAB were associated with more disability, more pain, and lower functioning [38, 39] and are also associated with increased psychological distress due to LBP, which has been identified as a contributing factor for higher pain levels and overall more disability. Psychological distress and suffering may worsen the pain levels and contribute to the development of common mental disorders, such as depression, anxiety, and stress-related disorders, which very often co-occur in patients with LBP. Depression is not only associated with pain but also with future and current work disability, as a negative outlook towards future work ability may influence the work ability scores [57]. Furthermore, a recent systematic review also showed that depression has an influence on LBP prognosis in both the acute and the subacute phases [58]. The present fear of worsening pain leads patients to movement avoidance or become overly careful leading to inactivity and deconditioning [24, 25, 27]. These periods of inactivity lead to pain and tiredness during movements, which only increases fear and creates a vicious cycle of mobility decrease and increase of psychological complaints that consequently decrease the ADL. This was confirmed in a study involving patients with LBP without structural pathologies compared to healthy controls [26]. The study reported that patients with chronic LBP had lower activity patterns compared to controls, e.g. they did less steps, spent more time lying down during the day and had overall less standing time. The authors also concluded that the especially lower activity levels in the evening and night might indicate that patients use up all their capacity during the day and are left with less capacity for leisure time activities later in the day [26].

Sexual function and sexuality has already been shown to have various positive effects on health and well-being of people; however, chronic conditions such as LBP have a detrimental effect on sexual functioning and satisfaction [44, 45] but studies on sexual outcomes in LBP are rare. Firstly, this is connected to common underestimations of sexual outcomes and sexuality as important aspects to include in research, and secondly to the common misperception that patients or study participants are put off by such questions [46]; however, the importance of these issues is seen across studies that all show high prevalence of sexual dissatisfaction and decrease of sexual frequency.

### Implications for therapy and rehabilitation

There is no universally accepted treatment or rehabilitation modality of non-specific LBP with general recommendations that patients should stay engaged in physical activity; however, not one single exercise program or method of rehabilitation is considered to be optimal for all patients, which is related to the multifactorial nature of LBP and complexities regarding etiology. A recent review on the effects of exercise and physical activity for nonspecific LBP reported that programs combining strength and flexibility exercises with aerobic training would be most beneficial [59]. Strengthening core musculature helps support the lumbar region of the spine, while improvements in flexibility of spinal tendons will increase the range of motion. Aerobic activity overall improves circulation reducing stiffness and reduces inflammation. By doing so physical activity prevents potential deconditioning, improves functionality and reduces pain, all of which help to reduce psychological distress and depression [59, 60]; however, these studies clearly indicate a need for a multimodal and multidisciplinary approach to LBP treatment, with more emphasis on the social and psychological aspects rather than just a biomechanical one. Studies therefore suggest that interventions aimed at people reporting LBP need to focus on the suffering (which was shown to be a potential mediator between depression and functional disability) and target the individual patients perceptions and beliefs on pain [61]; however, levels of physical activity remain low in most patients with LBP, and a potential way to introduce these is through workplace-based interventions. A Denmark-based study presented the results of a 12-week workplace intervention for nursing aids (pragmatic stepped wedge cluster randomised controlled trial) with various therapeutic modalities focusing on participatory ergonomics, physical training and cognitive behavioral training with 594 participants with LBP (mean age 47±10.2 years; 93% female) [57]. The results showed that such an intervention significantly reduced FABs. Furthermore, there was less kinesophobia, improved muscle strength, aerobic fitness and overall physical capacity. Even as the working capacity increased and demands on the worker decreased, the study reported a lack of effect on work ability. This may be linked to a high complexity of work ability that is influenced by various factors (e.g. low autonomy, high demands, poor environment,). Therefore, a more tailored approach for work ability dimensions may be needed for future interventions. A recent overview of systematic reviews looking into workplace interventions for LBP categorized the most common interventions as workplace modification (lumbar support or use of assistive devices), educational interventions (movement training, handling techniques) and physical exercise interventions. The authors concluded that physical exercise interventions were the only kind of intervention that showed moderate quality evidence alone or in combination with educational interventions. Other types of interventions were not found to be consistently effective. The authors concluded that multidimensional strategies that involve more than one approach to interventions seem to be most effective and the rationale behind them lies in the multidimensional etiology of LBP. Further evaluations of various workplace interventions are needed [62].

Studies looking into sexual outcomes and LBP underline the need of counselling patients with LBP on sexual function and sexual health. Sexual counselling should be offered to patients with LBP and therapeutic measures to patients with serious sexual maladaptation [46, 48, 52, 57]; however, a number of studies investigating the communication about sexual functioning and sex between physicians and patients are rare. Studies that have tried to research these issues note personal and organizational barriers, such as lack of preparation, training, privacy and time needed to talk about intimate issues with patients. Moreover, studies indicate that patients, nonetheless expect physicians to provide information about sex and talk openly about these issues in a supportive and educational manner that will relieve fear and provide more optimism [49]. A Moroccan cross-sectional study of patients experiencing LBP showed that even though 81% experienced sexual problems, 66% never discussed the subject with a physician. Furthermore, 94% expressed the need for management of sexual functioning issues associated with chronic LBP and 74% expected information and advice mostly on how to avoid pain, with 33% also agreeing that the partners should be included in these consultations [49]. Studies also showed that a high proportion of patients expect to be actively asked about sexual functioning and be given advice on it by healthcare providers. This may be problematic when most healthcare providers do not feel educated enough to provide such counselling. Therefore, as many of the authors of the included studies in this review, we also call for research in the field of sexual functioning with LBP that would ultimately lead to guidelines and educational material that healthcare practitioners may use in the daily routine.

### Strengths and limitations

A strength of the review lies in the combination of functional issues of everyday performance included: ADL, work ability and sexual function. Another strength is that potential modifying factors that are common to all three outcomes were investigated. To the best of our knowledge, this has not been reported so far. Furthermore, by including studies from different cultural and geographical settings it is shown that LBP is a global issue that generates similar problems for the patients. The major limitation also lies in the reviewed papers. Most of the included studies were observational non-randomized studies as well as cross-sectional studies done with relatively small samples, which prevent conclusions of causality. Conceptual issues are also common among the very few studies investigating sexual function and LBP. Some studies used extrapolation of healthy participants to derive the potential biomechanics during coitus in patients with LBP. Also, studies focused on sexual outcomes in couples where one partner reported LBP, but in light of the high prevalence of LBP studies should also focus on measurements and recommendations in situations where both partners experience LBP. Moreover, studies have investigated sexual satisfaction and function solely on heterosexual samples and vaginal sex and have highlighted that even minimal changes in the sexual positioning influence the biomechanics [53]. This would imply that there might be implications for non-heterosexual couples and practices outside vaginal sex. In addition, when examining sexual satisfaction measures of other sexual practices such as petting, fondling, masturbation need to be included in order to get a more comprehensive picture.

## Conclusion

Evidence decisively shows that LBP affects ADL, work ability and sexual functioning. At the core of these problems are the FAB that hindering movement and activity prevents the patients in achieving their full functional capacity even with LBP. Additional factors mediating the association between LBP and problems in everyday performance include deconditioning and common mental disorders. Physical training, comprehensive patient education, and workplace or home modification positively influence the factors mediating the association between LBP and everyday performance and are also beneficial for LBP itself. Therefore, they are promising factors that should be considered in routine treatment and rehabilitation in patients with LBP. Further research is necessary, not only to elucidate the etiology of LBP, but also in multimodal interventions in the management of LBP in both home and working environments. Issues surrounding sexuality and sexual functioning need to be further investigated as the few studies that have been published indicate a high prevalence of sexuality related problems.
